# A Case of Type 2 Diastematomyelia With Spina Bifida in a Pediatric Female Patient

**DOI:** 10.7759/cureus.55197

**Published:** 2024-02-28

**Authors:** Shivani S Bothara, Pratap Parihar

**Affiliations:** 1 Radiodiagnosis, Jawaharlal Nehru Medical College, Datta Meghe Institute of Higher Education & Research, Wardha, IND

**Keywords:** vancouver classification system, surgical intervention, multidisciplinary management, pediatric, congenital spinal anomalies, spina bifida, type 2 diastematomyelia

## Abstract

This case report presents the clinical and radiological findings of a seven-year-old female with type 2 diastematomyelia and spina bifida, emphasizing the complexity of congenital spinal anomalies in pediatric patients. The patient presented with a two-month history of lower back pain, prompting diagnostic investigations. Radiographic examination revealed spina bifida at the L3-L5 levels, subsequently confirmed by magnetic resonance imaging (MRI), which disclosed bifid spinous processes, an absent posterior arch, and a split spinal cord terminating at the L3-L4 disc levels. The Vancouver classification system facilitated a standardized characterization of congenital spinal anomalies. The multidisciplinary approach involving orthopedic and neurosurgical specialists led to a conclusive diagnosis of type 2 diastematomyelia with simple spinal dysraphism. Surgical intervention, encompassing laminectomy and correction of the split spinal cord, was successfully performed, resulting in the stabilization of the patient. This case underscores the importance of early diagnosis, advanced imaging modalities, and collaborative management in addressing rare congenital spinal anomalies. The discussion delves into the clinical implications, diagnostic challenges, and the pivotal role of surgical intervention. Insights from this case contribute to the existing literature, guiding healthcare professionals in understanding and managing similar cases with potential implications for future research and treatment strategies.

## Introduction

Spina bifida, a congenital neural tube defect, manifests as an incomplete closure of the vertebral arch during embryonic development, resulting in varying degrees of spinal cord malformation. Type 2 diastematomyelia, a rare subtype, involves the division of the spinal cord into two hemi-cords, often coexisting with spina bifida. This complex congenital anomaly poses diagnostic challenges and necessitates a multidisciplinary approach for effective management. The prevalence of spina bifida and diastematomyelia varies globally, with reported rates ranging from 0.1 to 0.5 per 1000 live births, making it a relatively rare condition [1]. The pathogenesis involves disturbances in neurulation during early embryonic development [2]. These anomalies can have significant clinical implications, including neurological deficits, musculoskeletal deformities, and lower back pain.

The clinical presentation of diastematomyelia can be diverse, ranging from asymptomatic cases to those with severe neurological deficits. Lower back pain in pediatric patients may raise suspicion, leading to diagnostic investigations such as radiography and magnetic resonance imaging (MRI) for a comprehensive evaluation [3]. In the presented case, a seven-year-old female with lower back pain underwent radiographic and MRI examinations, revealing spina bifida and diastematomyelia. The Vancouver classification system, commonly used in spine-related research, aids in characterizing and categorizing congenital spinal anomalies [4].

Surgical intervention is pivotal in managing diastematomyelia, addressing neurological symptoms, preventing further complications, and improving the patient's overall quality of life [5]. Laminectomy and correction of the split spinal cord, as performed in this case, represent established surgical techniques for managing this complex anomaly [6]. This case report aims to contribute to the existing literature on the clinical presentation, diagnostic approach, and successful surgical management of type 2 diastematomyelia with spina bifida in a pediatric patient. The insights gained from this case may aid healthcare professionals in understanding and addressing similar cases, ultimately improving patient outcomes.

## Case presentation

**Figure 1:**
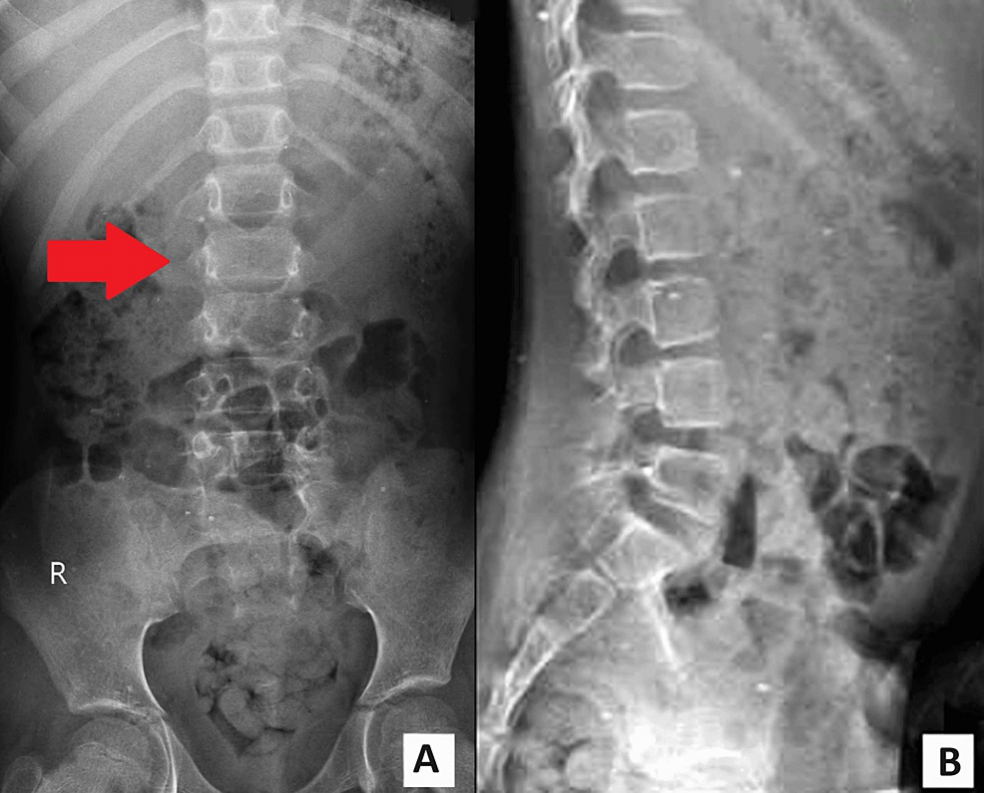
A and B show the X-Ray lumbar spine (AP and lateral) showing the bifid spinous process at L3, L4, and L5 levels AP: Anteroposterior

A seven-year-old female presented to the pediatric orthopedics clinic with a two-month history of lower back pain. The patient's parents reported the presence of a skin defect on the lower lumbar region since birth, prompting concern about a potential underlying spinal abnormality. The patient has no past medical or surgical history. Upon examination, the patient exhibited tenderness over the lower lumbar region. A visible skin defect was noted, consistent with the parents' report. Neurological examination revealed no motor deficits, but sensory abnormalities corresponding to the affected dermatomes were observed. To investigate the underlying cause further, the orthopedics department recommended a radiograph of the lumbar spine with anteroposterior (AP) and lateral views. The radiograph revealed a failure to close the vertebral arch at the L3-L5 levels, suggesting spina bifida (Figure 1).

**Figure 2:**
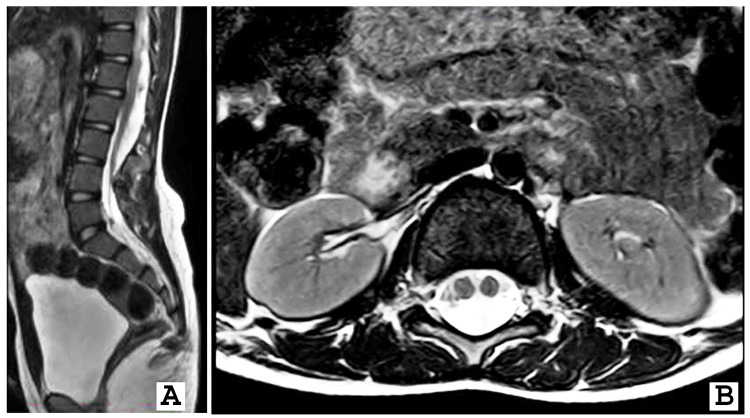
A and B, magnetic resonance imaging showing T2WI image with two spinal cords within the same dural sac-type 2 diastematomyelia at all L3-L5 levels T2WI: T2 weighted imaging

Subsequent MRI was performed to obtain a detailed assessment of the spinal cord and associated structures. The MRI revealed bifid spinous processes at the L3, L4, and L5 levels. The posterior arch was found to be absent in the sacral region. The spinal cord appeared low-lying, with the conus medullaris terminating at the L3-L4 disc levels. Additionally, the spinal cord split into two without duplicating the dural sac from the L1 to L4 disc levels, with incomplete fibrous septa noted at the L3-L4 disc levels (Figure 2). Based on the clinical-radiological correlation, the patient was diagnosed with type 2 diastematomyelia with simple spinal dysraphism, compounded by the presence of spina bifida.

Given the diagnosis, the patient underwent a laminectomy and correction of the split spinal cord. The surgical procedure was uneventful, and the patient showed satisfactory postoperative recovery. The patient's lower back pain resolved postoperatively, and a neurological examination revealed no new deficits.

## Discussion

The presented case of a seven-year-old female with type 2 diastematomyelia and spina bifida highlights the complexity of congenital spinal anomalies and the significance of multidisciplinary management. The clinical presentation of type 2 diastematomyelia often includes lower back pain, as observed in our patient. Although rare in pediatric populations, this symptom prompted further investigation, identifying spina bifida on radiography and subsequent detailed characterization through MRI [7]. The Vancouver classification system, commonly used in spine research, aids in standardizing congenital spinal anomalies, providing a comprehensive framework for clinicians and researchers [8].

The association of spina bifida and diastematomyelia underscores the complex interplay of genetic and environmental factors during embryonic development [9]. Understanding these pathogenetic mechanisms is crucial for improved prenatal screening and genetic counseling. Recent advancements in genetic research may contribute to unraveling the intricate etiology of these congenital anomalies [10]. The diagnostic challenge lies in the variability of clinical presentations and the need for advanced imaging modalities. The MRI, in our case, allowed for a detailed assessment of the spinal cord, revealing the bifid spinous processes, absent posterior arch, and the characteristic split of the spinal cord with incomplete fibrous septa [11]. Early and accurate diagnosis is paramount for timely intervention, preventing further neurological compromise.

Surgical management remains the cornerstone of treatment for type 2 diastematomyelia. Laminectomy and correction of the split spinal cord, as performed in this case, aim to alleviate neurological symptoms and prevent complications. Successful outcomes are contingent on the expertise of a multidisciplinary team, including orthopedic and neurosurgical specialists [12]. Regular follow-up is essential to monitor the patient's stability and address any potential complications that may arise over time. The rarity of type 2 diastematomyelia necessitates collaboration among healthcare professionals to consolidate knowledge and enhance treatment strategies. Ongoing research is crucial to refining diagnostic approaches, elucidating underlying genetic factors, and exploring novel therapeutic modalities. Long-term studies are warranted to assess the durability of surgical outcomes and the patient's quality of life throughout their developmental stages.

## Conclusions

In conclusion, this case emphasizes the crucial role of early diagnosis and surgical intervention in treating type 2 diastematomyelia with spina bifida in pediatric patients. The initial complaints of lower back pain and a congenital skin defect prompted thorough radiological examinations, leading to the successful execution of laminectomy and correction of the split spinal cord. This multidisciplinary approach, involving orthopedic and neurosurgical specialists, resulted in postoperative stability and symptom resolution. The case underscores the significance of timely intervention in improving outcomes and quality of life for affected individuals. Further research is needed to refine treatment approaches, and collaborative efforts among healthcare professionals remain essential in advancing our understanding of these complex congenital anomalies. Overall, this case contributes valuable insights to the existing literature, highlighting the positive impact of comprehensive medical care and surgical expertise on the well-being of pediatric patients with type 2 diastematomyelia.
